# Supplementary material for: A Classification Approach for Cancer Survivors from Those Cancer-Free, Based on Health Behaviors: Analysis of the Lifelines Cohort
Source: Cancers (Basel). 2021 May 12;13(10):2335. doi: 10.3390/cancers13102335 (PMC8151639; doi:10.3390/cancers13102335)

Supplementary file 1. Heat map of the consistency on feature importance in the random forest classifier by the MDG for every subset, a more saturated colour means a more important feature in the analysis. The heat maps show the consistency when including all features (a), when including only health-risk behaviours (b), and in the case-control analysis (c).

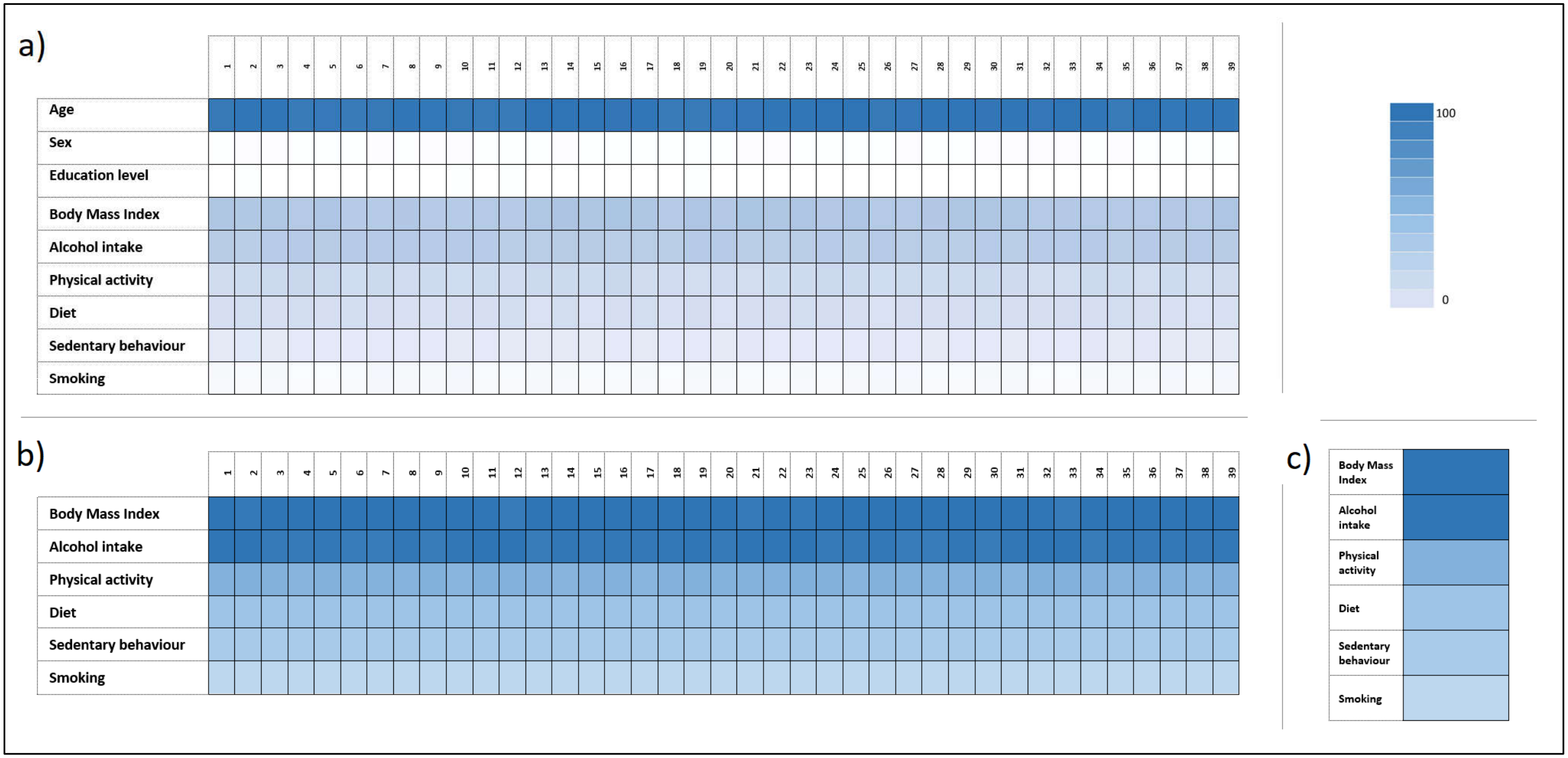

Supplement: Supplementary file 1 [file cancers-13-02335-s001.zip › cancers-1175173-supplementary.pdf]
